# Supplementary material for: Modeling glioblastoma heterogeneity as a dynamic network of cell states
Source: Mol Syst Biol. 2021 Sep 16;17(9):e10105. doi: 10.15252/msb.202010105 (PMC8444284; doi:10.15252/msb.202010105)
Supplement: Supplementary file 6 — Source Data for Figure 5 [file MSB-17-e10105-s004.zip › Figure5A_sourcedata/GSEA_3071/nosigdb_stateIV.GseaPreranked.1622795164626/DOROSZKO_U3054MG_CLUSTER_4.html]

Details for gene set DOROSZKO\_U3054MG\_CLUSTER\_4[GSEA]

|  || Dataset | state53071 |
| Phenotype | NoPhenotypeAvailable |
| Upregulated in class | na\_neg |
| GeneSet | DOROSZKO\_U3054MG\_CLUSTER\_4 |
| Enrichment Score (ES) | -0.55169886 |
| Normalized Enrichment Score (NES) | -3.6203198 |
| Nominal p-value | 0.0 |
| FDR q-value | 0.0 |
| FWER p-Value | 0.0 |
Table: GSEA Results Summary

  

Fig 1: Enrichment plot: DOROSZKO\_U3054MG\_CLUSTER\_4      
 Profile of the Running ES Score & Positions of GeneSet Members on the Rank Ordered List

  

| PROBE | GENE SYMBOL | GENE\_TITLE | RANK IN GENE LIST | RANK METRIC SCORE | RUNNING ES | CORE ENRICHMENT || 1 | PMP2 |  |  | 11 | 2.494 | 0.0058 | No |
| 2 | MCUB |  |  | 150 | 1.375 | -0.0184 | No |
| 3 | CDCA7L |  |  | 185 | 1.292 | -0.0212 | No |
| 4 | FBLN1 |  |  | 213 | 1.226 | -0.0229 | No |
| 5 | DRAXIN |  |  | 219 | 1.210 | -0.0200 | No |
| 6 | TCEAL7 |  |  | 249 | 1.150 | -0.0223 | No |
| 7 | HES6 |  |  | 250 | 1.150 | -0.0186 | No |
| 8 | HUNK |  |  | 251 | 1.146 | -0.0149 | No |
| 9 | PDLIM4 |  |  | 435 | 0.867 | -0.0500 | No |
| 10 | PPP1R14C |  |  | 596 | 0.729 | -0.0807 | No |
| 11 | EMP2 |  |  | 732 | 0.642 | -0.1066 | No |
| 12 | CDCA7 |  |  | 818 | 0.596 | -0.1222 | No |
| 13 | OLFM1 |  |  | 1064 | 0.502 | -0.1713 | No |
| 14 | SYNE2 |  |  | 1094 | 0.493 | -0.1757 | No |
| 15 | VMA21 |  |  | 1134 | 0.484 | -0.1822 | No |
| 16 | ASRGL1 |  |  | 1258 | 0.451 | -0.2062 | No |
| 17 | RSRC1 |  |  | 1347 | 0.428 | -0.2230 | No |
| 18 | ACTL6A |  |  | 1789 | 0.333 | -0.3132 | No |
| 19 | OXCT1 |  |  | 1892 | 0.315 | -0.3333 | No |
| 20 | NABP2 |  |  | 1985 | 0.300 | -0.3513 | No |
| 21 | RBBP4 |  |  | 2000 | 0.297 | -0.3533 | No |
| 22 | RNF168 |  |  | 2114 | 0.282 | -0.3757 | No |
| 23 | BID |  |  | 2118 | 0.281 | -0.3754 | No |
| 24 | RTN3 |  |  | 2160 | 0.274 | -0.3830 | No |
| 25 | CARHSP1 |  |  | 2185 | 0.270 | -0.3871 | No |
| 26 | GART |  |  | 2337 | -0.251 | -0.4176 | No |
| 27 | CCDC137 |  |  | 2378 | -0.256 | -0.4250 | No |
| 28 | IFRD2 |  |  | 2399 | -0.258 | -0.4283 | No |
| 29 | CTDSPL2 |  |  | 2410 | -0.259 | -0.4295 | No |
| 30 | POLD1 |  |  | 2417 | -0.260 | -0.4300 | No |
| 31 | DUS1L |  |  | 2424 | -0.260 | -0.4304 | No |
| 32 | MRM2 |  |  | 2436 | -0.262 | -0.4318 | No |
| 33 | SPIN4 |  |  | 2465 | -0.265 | -0.4367 | No |
| 34 | PTTG1 |  |  | 2470 | -0.266 | -0.4367 | No |
| 35 | SHMT1 |  |  | 2478 | -0.267 | -0.4373 | No |
| 36 | LAGE3 |  |  | 2483 | -0.268 | -0.4372 | No |
| 37 | MRPL12 |  |  | 2484 | -0.268 | -0.4364 | No |
| 38 | LMNB1 |  |  | 2487 | -0.268 | -0.4359 | No |
| 39 | ZNF93 |  |  | 2507 | -0.270 | -0.4390 | No |
| 40 | RAD1 |  |  | 2544 | -0.274 | -0.4456 | No |
| 41 | RBFA |  |  | 2580 | -0.280 | -0.4519 | No |
| 42 | THOP1 |  |  | 2591 | -0.281 | -0.4531 | No |
| 43 | RNASEH2A |  |  | 2595 | -0.282 | -0.4528 | No |
| 44 | BAZ1A |  |  | 2612 | -0.284 | -0.4552 | No |
| 45 | CMC2 |  |  | 2632 | -0.287 | -0.4582 | No |
| 46 | POP7 |  |  | 2651 | -0.290 | -0.4610 | No |
| 47 | MRPS11 |  |  | 2685 | -0.293 | -0.4669 | No |
| 48 | FN3KRP |  |  | 2701 | -0.295 | -0.4690 | No |
| 49 | PSMD1 |  |  | 2727 | -0.297 | -0.4732 | No |
| 50 | AHCY |  |  | 2734 | -0.297 | -0.4735 | No |
| 51 | TRIM28 |  |  | 2737 | -0.298 | -0.4730 | No |
| 52 | CCNE1 |  |  | 2741 | -0.299 | -0.4726 | No |
| 53 | CDC25A |  |  | 2753 | -0.301 | -0.4739 | No |
| 54 | BOLA3 |  |  | 2755 | -0.301 | -0.4732 | No |
| 55 | PSMG3 |  |  | 2765 | -0.302 | -0.4741 | No |
| 56 | VOPP1 |  |  | 2783 | -0.304 | -0.4766 | No |
| 57 | ZNF519 |  |  | 2790 | -0.305 | -0.4769 | No |
| 58 | TMEM237 |  |  | 2811 | -0.307 | -0.4800 | No |
| 59 | RAD54L |  |  | 2855 | -0.313 | -0.4879 | No |
| 60 | PANK2 |  |  | 2869 | -0.315 | -0.4896 | No |
| 61 | ARL6IP6 |  |  | 2877 | -0.316 | -0.4900 | No |
| 62 | RMI2 |  |  | 2893 | -0.318 | -0.4921 | No |
| 63 | DCK |  |  | 2907 | -0.319 | -0.4937 | No |
| 64 | EME1 |  |  | 2913 | -0.320 | -0.4937 | No |
| 65 | PPAT |  |  | 2915 | -0.320 | -0.4929 | No |
| 66 | MCUR1 |  |  | 2924 | -0.321 | -0.4935 | No |
| 67 | EZH2 |  |  | 2946 | -0.324 | -0.4968 | No |
| 68 | NUP50 |  |  | 2954 | -0.326 | -0.4972 | No |
| 69 | RAD51D |  |  | 2956 | -0.327 | -0.4964 | No |
| 70 | CNTLN |  |  | 2960 | -0.327 | -0.4960 | No |
| 71 | TIMM10 |  |  | 2963 | -0.328 | -0.4953 | No |
| 72 | SMC1A |  |  | 2968 | -0.328 | -0.4951 | No |
| 73 | WDR54 |  |  | 2987 | -0.331 | -0.4977 | No |
| 74 | PHTF2 |  |  | 2993 | -0.332 | -0.4977 | No |
| 75 | GLO1 |  |  | 2996 | -0.332 | -0.4971 | No |
| 76 | HACD3 |  |  | 3013 | -0.334 | -0.4993 | No |
| 77 | SMC4 |  |  | 3035 | -0.337 | -0.5025 | No |
| 78 | MSH6 |  |  | 3051 | -0.339 | -0.5046 | No |
| 79 | C1orf35 |  |  | 3055 | -0.339 | -0.5041 | No |
| 80 | MCMBP |  |  | 3056 | -0.339 | -0.5030 | No |
| 81 | UQCC2 |  |  | 3062 | -0.340 | -0.5029 | No |
| 82 | PPM1G |  |  | 3071 | -0.341 | -0.5035 | No |
| 83 | SASS6 |  |  | 3091 | -0.344 | -0.5063 | No |
| 84 | HAUS1 |  |  | 3106 | -0.347 | -0.5081 | No |
| 85 | EIF4A3 |  |  | 3116 | -0.349 | -0.5088 | No |
| 86 | SLC2A4RG |  |  | 3122 | -0.349 | -0.5087 | No |
| 87 | ACD |  |  | 3124 | -0.350 | -0.5078 | No |
| 88 | NUP205 |  |  | 3125 | -0.350 | -0.5067 | No |
| 89 | RPA1 |  |  | 3127 | -0.350 | -0.5058 | No |
| 90 | NUP188 |  |  | 3161 | -0.355 | -0.5115 | No |
| 91 | ORC1 |  |  | 3190 | -0.358 | -0.5161 | No |
| 92 | PSMD14 |  |  | 3196 | -0.359 | -0.5160 | No |
| 93 | SRSF10 |  |  | 3223 | -0.364 | -0.5202 | No |
| 94 | TEDC1 |  |  | 3239 | -0.366 | -0.5221 | No |
| 95 | MCM2 |  |  | 3240 | -0.366 | -0.5209 | No |
| 96 | PDHA1 |  |  | 3244 | -0.367 | -0.5204 | No |
| 97 | C5orf34 |  |  | 3251 | -0.368 | -0.5204 | No |
| 98 | SIGMAR1 |  |  | 3253 | -0.369 | -0.5195 | No |
| 99 | POC1A |  |  | 3305 | -0.376 | -0.5288 | No |
| 100 | PKMYT1 |  |  | 3311 | -0.377 | -0.5286 | No |
| 101 | MXD3 |  |  | 3316 | -0.377 | -0.5282 | No |
| 102 | CISD2 |  |  | 3319 | -0.378 | -0.5274 | No |
| 103 | HIRIP3 |  |  | 3328 | -0.379 | -0.5279 | No |
| 104 | POLE |  |  | 3329 | -0.379 | -0.5266 | No |
| 105 | NUP107 |  |  | 3342 | -0.380 | -0.5279 | No |
| 106 | DIAPH1 |  |  | 3354 | -0.382 | -0.5289 | No |
| 107 | CPSF4 |  |  | 3360 | -0.383 | -0.5287 | No |
| 108 | TMEM38B |  |  | 3361 | -0.384 | -0.5275 | No |
| 109 | PCNT |  |  | 3374 | -0.387 | -0.5287 | No |
| 110 | CHEK2 |  |  | 3396 | -0.390 | -0.5318 | No |
| 111 | DCTPP1 |  |  | 3414 | -0.393 | -0.5341 | No |
| 112 | RAD51C |  |  | 3415 | -0.393 | -0.5328 | No |
| 113 | ZGRF1 |  |  | 3417 | -0.394 | -0.5318 | No |
| 114 | CYB5A |  |  | 3418 | -0.394 | -0.5305 | No |
| 115 | STIP1 |  |  | 3427 | -0.395 | -0.5309 | No |
| 116 | HAUS8 |  |  | 3443 | -0.398 | -0.5327 | No |
| 117 | CHAF1B |  |  | 3451 | -0.400 | -0.5329 | No |
| 118 | MNS1 |  |  | 3455 | -0.401 | -0.5322 | No |
| 119 | SKP2 |  |  | 3472 | -0.404 | -0.5342 | No |
| 120 | GEN1 |  |  | 3494 | -0.408 | -0.5372 | No |
| 121 | CENPX |  |  | 3495 | -0.409 | -0.5359 | No |
| 122 | ZNF367 |  |  | 3507 | -0.411 | -0.5369 | No |
| 123 | CDK2 |  |  | 3521 | -0.414 | -0.5382 | No |
| 124 | TFDP1 |  |  | 3526 | -0.415 | -0.5377 | No |
| 125 | DDX11 |  |  | 3527 | -0.415 | -0.5364 | No |
| 126 | POLE2 |  |  | 3540 | -0.417 | -0.5375 | No |
| 127 | MRPL14 |  |  | 3543 | -0.418 | -0.5366 | No |
| 128 | TIPIN |  |  | 3559 | -0.420 | -0.5383 | No |
| 129 | MRPL18 |  |  | 3568 | -0.422 | -0.5386 | No |
| 130 | GPD2 |  |  | 3589 | -0.426 | -0.5414 | No |
| 131 | PAICS |  |  | 3594 | -0.427 | -0.5409 | No |
| 132 | POLQ |  |  | 3600 | -0.429 | -0.5405 | No |
| 133 | CKAP2 |  |  | 3633 | -0.433 | -0.5457 | No |
| 134 | SNRPA1 |  |  | 3638 | -0.434 | -0.5452 | No |
| 135 | SPATA33 |  |  | 3660 | -0.437 | -0.5481 | No |
| 136 | RMI1 |  |  | 3668 | -0.438 | -0.5481 | No |
| 137 | TMEM106C |  |  | 3669 | -0.438 | -0.5467 | No |
| 138 | CEP57 |  |  | 3694 | -0.443 | -0.5503 | Yes |
| 139 | ACAT2 |  |  | 3695 | -0.443 | -0.5488 | Yes |
| 140 | CCDC138 |  |  | 3696 | -0.444 | -0.5474 | Yes |
| 141 | PRPS2 |  |  | 3700 | -0.445 | -0.5466 | Yes |
| 142 | FANCD2 |  |  | 3701 | -0.445 | -0.5452 | Yes |
| 143 | KDELC2 |  |  | 3706 | -0.447 | -0.5446 | Yes |
| 144 | RAD18 |  |  | 3717 | -0.448 | -0.5452 | Yes |
| 145 | BRI3BP |  |  | 3722 | -0.449 | -0.5446 | Yes |
| 146 | PGP |  |  | 3725 | -0.450 | -0.5435 | Yes |
| 147 | CBR3 |  |  | 3745 | -0.454 | -0.5460 | Yes |
| 148 | HAUS2 |  |  | 3746 | -0.455 | -0.5445 | Yes |
| 149 | TUBG1 |  |  | 3775 | -0.460 | -0.5488 | Yes |
| 150 | PRADC1 |  |  | 3777 | -0.461 | -0.5476 | Yes |
| 151 | GPN3 |  |  | 3790 | -0.464 | -0.5486 | Yes |
| 152 | PLK4 |  |  | 3791 | -0.465 | -0.5471 | Yes |
| 153 | DDIAS |  |  | 3795 | -0.465 | -0.5462 | Yes |
| 154 | DERA |  |  | 3799 | -0.466 | -0.5453 | Yes |
| 155 | YEATS4 |  |  | 3803 | -0.466 | -0.5444 | Yes |
| 156 | KIF18B |  |  | 3807 | -0.468 | -0.5435 | Yes |
| 157 | LMNB2 |  |  | 3817 | -0.469 | -0.5439 | Yes |
| 158 | FBL |  |  | 3819 | -0.469 | -0.5426 | Yes |
| 159 | CENPO |  |  | 3820 | -0.469 | -0.5411 | Yes |
| 160 | UFD1 |  |  | 3831 | -0.472 | -0.5416 | Yes |
| 161 | KIF22 |  |  | 3832 | -0.472 | -0.5401 | Yes |
| 162 | GINS4 |  |  | 3843 | -0.474 | -0.5407 | Yes |
| 163 | NUP155 |  |  | 3868 | -0.480 | -0.5441 | Yes |
| 164 | SUPT16H |  |  | 3872 | -0.481 | -0.5431 | Yes |
| 165 | TIMELESS |  |  | 3877 | -0.482 | -0.5424 | Yes |
| 166 | FKBP5 |  |  | 3899 | -0.487 | -0.5452 | Yes |
| 167 | CCND3 |  |  | 3901 | -0.487 | -0.5438 | Yes |
| 168 | ECT2 |  |  | 3902 | -0.487 | -0.5423 | Yes |
| 169 | PARP2 |  |  | 3910 | -0.490 | -0.5421 | Yes |
| 170 | SMCHD1 |  |  | 3911 | -0.490 | -0.5406 | Yes |
| 171 | MIS18BP1 |  |  | 3913 | -0.490 | -0.5392 | Yes |
| 172 | MRPL22 |  |  | 3921 | -0.492 | -0.5391 | Yes |
| 173 | POLA1 |  |  | 3931 | -0.494 | -0.5393 | Yes |
| 174 | ICMT |  |  | 3943 | -0.496 | -0.5400 | Yes |
| 175 | SRPK1 |  |  | 3944 | -0.496 | -0.5384 | Yes |
| 176 | NPW |  |  | 3949 | -0.497 | -0.5376 | Yes |
| 177 | SMC3 |  |  | 3952 | -0.498 | -0.5365 | Yes |
| 178 | CIP2A |  |  | 3968 | -0.502 | -0.5379 | Yes |
| 179 | NUP85 |  |  | 3970 | -0.503 | -0.5365 | Yes |
| 180 | MAGOHB |  |  | 3992 | -0.509 | -0.5392 | Yes |
| 181 | SLBP |  |  | 3994 | -0.509 | -0.5378 | Yes |
| 182 | CENPF |  |  | 4003 | -0.511 | -0.5378 | Yes |
| 183 | ARHGAP11A |  |  | 4007 | -0.513 | -0.5368 | Yes |
| 184 | HADH |  |  | 4009 | -0.514 | -0.5353 | Yes |
| 185 | HIST1H1E |  |  | 4011 | -0.515 | -0.5339 | Yes |
| 186 | CCDC15 |  |  | 4019 | -0.518 | -0.5337 | Yes |
| 187 | WDR34 |  |  | 4022 | -0.518 | -0.5324 | Yes |
| 188 | TOMM40 |  |  | 4030 | -0.520 | -0.5322 | Yes |
| 189 | YWHAH |  |  | 4032 | -0.521 | -0.5307 | Yes |
| 190 | PHF19 |  |  | 4041 | -0.523 | -0.5307 | Yes |
| 191 | SDF2L1 |  |  | 4049 | -0.525 | -0.5304 | Yes |
| 192 | NCAPD2 |  |  | 4055 | -0.526 | -0.5298 | Yes |
| 193 | WDR76 |  |  | 4060 | -0.527 | -0.5289 | Yes |
| 194 | MMS22L |  |  | 4062 | -0.528 | -0.5274 | Yes |
| 195 | ITGB3BP |  |  | 4071 | -0.531 | -0.5274 | Yes |
| 196 | CCHCR1 |  |  | 4078 | -0.533 | -0.5269 | Yes |
| 197 | RRP7A |  |  | 4079 | -0.533 | -0.5252 | Yes |
| 198 | ZWILCH |  |  | 4082 | -0.534 | -0.5239 | Yes |
| 199 | ACP1 |  |  | 4084 | -0.535 | -0.5224 | Yes |
| 200 | EXO1 |  |  | 4094 | -0.539 | -0.5225 | Yes |
| 201 | BLM |  |  | 4100 | -0.541 | -0.5218 | Yes |
| 202 | CCNB2 |  |  | 4103 | -0.542 | -0.5205 | Yes |
| 203 | E2F8 |  |  | 4104 | -0.542 | -0.5187 | Yes |
| 204 | BRIX1 |  |  | 4106 | -0.542 | -0.5172 | Yes |
| 205 | CACYBP |  |  | 4109 | -0.542 | -0.5158 | Yes |
| 206 | TTF2 |  |  | 4110 | -0.543 | -0.5141 | Yes |
| 207 | SSRP1 |  |  | 4112 | -0.544 | -0.5126 | Yes |
| 208 | PRPF4 |  |  | 4118 | -0.545 | -0.5118 | Yes |
| 209 | RFWD3 |  |  | 4122 | -0.546 | -0.5107 | Yes |
| 210 | TMEM243 |  |  | 4135 | -0.550 | -0.5114 | Yes |
| 211 | CENPJ |  |  | 4148 | -0.555 | -0.5121 | Yes |
| 212 | FOXM1 |  |  | 4158 | -0.556 | -0.5122 | Yes |
| 213 | NXT1 |  |  | 4170 | -0.560 | -0.5127 | Yes |
| 214 | KNTC1 |  |  | 4176 | -0.561 | -0.5119 | Yes |
| 215 | PPIF |  |  | 4178 | -0.561 | -0.5103 | Yes |
| 216 | HPRT1 |  |  | 4179 | -0.562 | -0.5085 | Yes |
| 217 | EXOSC9 |  |  | 4189 | -0.564 | -0.5085 | Yes |
| 218 | CENPQ |  |  | 4190 | -0.564 | -0.5067 | Yes |
| 219 | SPC24 |  |  | 4194 | -0.565 | -0.5055 | Yes |
| 220 | MRE11 |  |  | 4195 | -0.566 | -0.5037 | Yes |
| 221 | MRPL17 |  |  | 4210 | -0.569 | -0.5047 | Yes |
| 222 | CLN6 |  |  | 4214 | -0.570 | -0.5035 | Yes |
| 223 | PSMC3 |  |  | 4224 | -0.573 | -0.5036 | Yes |
| 224 | SOD3 |  |  | 4227 | -0.574 | -0.5021 | Yes |
| 225 | BLVRA |  |  | 4228 | -0.574 | -0.5003 | Yes |
| 226 | MIS18A |  |  | 4230 | -0.575 | -0.4986 | Yes |
| 227 | TCOF1 |  |  | 4231 | -0.575 | -0.4968 | Yes |
| 228 | COMMD4 |  |  | 4242 | -0.577 | -0.4970 | Yes |
| 229 | HMGB2 |  |  | 4243 | -0.578 | -0.4951 | Yes |
| 230 | BUB1 |  |  | 4246 | -0.578 | -0.4937 | Yes |
| 231 | BUB3 |  |  | 4249 | -0.580 | -0.4922 | Yes |
| 232 | MORC4 |  |  | 4251 | -0.581 | -0.4906 | Yes |
| 233 | EEF1E1 |  |  | 4257 | -0.583 | -0.4897 | Yes |
| 234 | FANCB |  |  | 4259 | -0.584 | -0.4881 | Yes |
| 235 | PPIH |  |  | 4268 | -0.587 | -0.4878 | Yes |
| 236 | ARPC5L |  |  | 4269 | -0.588 | -0.4859 | Yes |
| 237 | MCM6 |  |  | 4273 | -0.590 | -0.4846 | Yes |
| 238 | MTHFD1 |  |  | 4274 | -0.590 | -0.4827 | Yes |
| 239 | CAD |  |  | 4289 | -0.595 | -0.4837 | Yes |
| 240 | SPAG5 |  |  | 4290 | -0.595 | -0.4818 | Yes |
| 241 | EXOSC8 |  |  | 4293 | -0.596 | -0.4803 | Yes |
| 242 | SLF1 |  |  | 4294 | -0.596 | -0.4784 | Yes |
| 243 | RAB5IF |  |  | 4297 | -0.597 | -0.4769 | Yes |
| 244 | FARSB |  |  | 4299 | -0.597 | -0.4752 | Yes |
| 245 | BRIP1 |  |  | 4301 | -0.598 | -0.4735 | Yes |
| 246 | MAP3K20 |  |  | 4308 | -0.599 | -0.4728 | Yes |
| 247 | ERCC6L |  |  | 4310 | -0.600 | -0.4710 | Yes |
| 248 | UPF3B |  |  | 4320 | -0.602 | -0.4710 | Yes |
| 249 | KIF15 |  |  | 4324 | -0.603 | -0.4696 | Yes |
| 250 | ASF1B |  |  | 4328 | -0.606 | -0.4683 | Yes |
| 251 | POLE3 |  |  | 4336 | -0.610 | -0.4678 | Yes |
| 252 | ATIC |  |  | 4338 | -0.611 | -0.4660 | Yes |
| 253 | SGO1 |  |  | 4339 | -0.611 | -0.4641 | Yes |
| 254 | RFC4 |  |  | 4340 | -0.611 | -0.4621 | Yes |
| 255 | RCC1 |  |  | 4341 | -0.612 | -0.4601 | Yes |
| 256 | POLE4 |  |  | 4360 | -0.621 | -0.4619 | Yes |
| 257 | KPNA2 |  |  | 4361 | -0.621 | -0.4599 | Yes |
| 258 | KNL1 |  |  | 4367 | -0.624 | -0.4589 | Yes |
| 259 | LIG1 |  |  | 4368 | -0.624 | -0.4569 | Yes |
| 260 | RBBP8 |  |  | 4375 | -0.627 | -0.4561 | Yes |
| 261 | POLA2 |  |  | 4379 | -0.627 | -0.4547 | Yes |
| 262 | DNMT1 |  |  | 4388 | -0.632 | -0.4543 | Yes |
| 263 | TPRKB |  |  | 4399 | -0.635 | -0.4544 | Yes |
| 264 | FANCA |  |  | 4403 | -0.635 | -0.4529 | Yes |
| 265 | ALYREF |  |  | 4406 | -0.638 | -0.4513 | Yes |
| 266 | FIGNL1 |  |  | 4411 | -0.639 | -0.4501 | Yes |
| 267 | CSE1L |  |  | 4419 | -0.642 | -0.4494 | Yes |
| 268 | CEP78 |  |  | 4420 | -0.642 | -0.4474 | Yes |
| 269 | KIF14 |  |  | 4424 | -0.643 | -0.4459 | Yes |
| 270 | C19orf48 |  |  | 4427 | -0.644 | -0.4443 | Yes |
| 271 | DONSON |  |  | 4444 | -0.652 | -0.4455 | Yes |
| 272 | SAC3D1 |  |  | 4462 | -0.660 | -0.4469 | Yes |
| 273 | CENPS |  |  | 4465 | -0.661 | -0.4452 | Yes |
| 274 | DDX39A |  |  | 4467 | -0.661 | -0.4432 | Yes |
| 275 | RBL1 |  |  | 4469 | -0.662 | -0.4413 | Yes |
| 276 | SHCBP1 |  |  | 4473 | -0.667 | -0.4398 | Yes |
| 277 | EIF4EBP1 |  |  | 4474 | -0.667 | -0.4376 | Yes |
| 278 | RPA3 |  |  | 4482 | -0.670 | -0.4369 | Yes |
| 279 | BUB1B |  |  | 4483 | -0.670 | -0.4348 | Yes |
| 280 | MASTL |  |  | 4490 | -0.673 | -0.4339 | Yes |
| 281 | PSMB9 |  |  | 4494 | -0.677 | -0.4323 | Yes |
| 282 | H2AFX |  |  | 4495 | -0.677 | -0.4301 | Yes |
| 283 | NUDT15 |  |  | 4506 | -0.684 | -0.4300 | Yes |
| 284 | SNRPF |  |  | 4514 | -0.687 | -0.4292 | Yes |
| 285 | DHFR |  |  | 4517 | -0.688 | -0.4274 | Yes |
| 286 | NCAPH2 |  |  | 4534 | -0.698 | -0.4285 | Yes |
| 287 | MND1 |  |  | 4538 | -0.699 | -0.4269 | Yes |
| 288 | RFC5 |  |  | 4543 | -0.701 | -0.4254 | Yes |
| 289 | NOP56 |  |  | 4556 | -0.708 | -0.4256 | Yes |
| 290 | CCNE2 |  |  | 4564 | -0.714 | -0.4248 | Yes |
| 291 | DTYMK |  |  | 4566 | -0.715 | -0.4227 | Yes |
| 292 | NCAPG |  |  | 4568 | -0.716 | -0.4206 | Yes |
| 293 | DBF4 |  |  | 4570 | -0.717 | -0.4185 | Yes |
| 294 | EBP |  |  | 4571 | -0.718 | -0.4162 | Yes |
| 295 | CTPS1 |  |  | 4572 | -0.718 | -0.4139 | Yes |
| 296 | MGME1 |  |  | 4573 | -0.722 | -0.4115 | Yes |
| 297 | IKBIP |  |  | 4584 | -0.727 | -0.4113 | Yes |
| 298 | PRIM2 |  |  | 4585 | -0.728 | -0.4089 | Yes |
| 299 | RFC2 |  |  | 4588 | -0.729 | -0.4070 | Yes |
| 300 | PBK |  |  | 4593 | -0.730 | -0.4055 | Yes |
| 301 | C9orf40 |  |  | 4596 | -0.732 | -0.4035 | Yes |
| 302 | CEP152 |  |  | 4599 | -0.733 | -0.4016 | Yes |
| 303 | CKS2 |  |  | 4600 | -0.733 | -0.3992 | Yes |
| 304 | SVIP |  |  | 4609 | -0.737 | -0.3985 | Yes |
| 305 | STIL |  |  | 4615 | -0.740 | -0.3972 | Yes |
| 306 | SPDL1 |  |  | 4617 | -0.741 | -0.3950 | Yes |
| 307 | BIRC5 |  |  | 4618 | -0.745 | -0.3926 | Yes |
| 308 | CKLF |  |  | 4619 | -0.746 | -0.3902 | Yes |
| 309 | RACGAP1 |  |  | 4623 | -0.748 | -0.3884 | Yes |
| 310 | PARPBP |  |  | 4625 | -0.749 | -0.3862 | Yes |
| 311 | E2F1 |  |  | 4637 | -0.760 | -0.3860 | Yes |
| 312 | RPA2 |  |  | 4638 | -0.760 | -0.3836 | Yes |
| 313 | NCAPD3 |  |  | 4640 | -0.760 | -0.3814 | Yes |
| 314 | DKC1 |  |  | 4646 | -0.763 | -0.3799 | Yes |
| 315 | USP1 |  |  | 4649 | -0.763 | -0.3779 | Yes |
| 316 | SGO2 |  |  | 4650 | -0.764 | -0.3754 | Yes |
| 317 | NUSAP1 |  |  | 4654 | -0.765 | -0.3736 | Yes |
| 318 | SDC1 |  |  | 4663 | -0.770 | -0.3728 | Yes |
| 319 | NT5C3A |  |  | 4664 | -0.771 | -0.3703 | Yes |
| 320 | FANCI |  |  | 4665 | -0.772 | -0.3678 | Yes |
| 321 | GGCT |  |  | 4666 | -0.772 | -0.3653 | Yes |
| 322 | SLC43A3 |  |  | 4668 | -0.775 | -0.3630 | Yes |
| 323 | MSH2 |  |  | 4673 | -0.781 | -0.3613 | Yes |
| 324 | CENPH |  |  | 4679 | -0.786 | -0.3598 | Yes |
| 325 | MIF |  |  | 4692 | -0.790 | -0.3598 | Yes |
| 326 | COPS3 |  |  | 4694 | -0.791 | -0.3574 | Yes |
| 327 | MYBL1 |  |  | 4697 | -0.795 | -0.3553 | Yes |
| 328 | CDC45 |  |  | 4702 | -0.798 | -0.3536 | Yes |
| 329 | CENPL |  |  | 4703 | -0.799 | -0.3510 | Yes |
| 330 | MCM7 |  |  | 4704 | -0.800 | -0.3484 | Yes |
| 331 | SAE1 |  |  | 4711 | -0.801 | -0.3471 | Yes |
| 332 | WDHD1 |  |  | 4713 | -0.803 | -0.3447 | Yes |
| 333 | TCF19 |  |  | 4719 | -0.807 | -0.3431 | Yes |
| 334 | TRIP13 |  |  | 4721 | -0.808 | -0.3407 | Yes |
| 335 | GTSE1 |  |  | 4722 | -0.808 | -0.3381 | Yes |
| 336 | KIF4A |  |  | 4727 | -0.812 | -0.3364 | Yes |
| 337 | ATAD3A |  |  | 4729 | -0.814 | -0.3339 | Yes |
| 338 | SHMT2 |  |  | 4737 | -0.820 | -0.3328 | Yes |
| 339 | KIF20B |  |  | 4745 | -0.825 | -0.3316 | Yes |
| 340 | CDCA8 |  |  | 4747 | -0.827 | -0.3291 | Yes |
| 341 | CHAF1A |  |  | 4751 | -0.828 | -0.3271 | Yes |
| 342 | POLD3 |  |  | 4752 | -0.830 | -0.3244 | Yes |
| 343 | MELK |  |  | 4758 | -0.835 | -0.3227 | Yes |
| 344 | KIF11 |  |  | 4760 | -0.835 | -0.3202 | Yes |
| 345 | CHEK1 |  |  | 4764 | -0.837 | -0.3182 | Yes |
| 346 | C21orf58 |  |  | 4767 | -0.838 | -0.3159 | Yes |
| 347 | CKAP2L |  |  | 4769 | -0.839 | -0.3134 | Yes |
| 348 | VRK1 |  |  | 4773 | -0.842 | -0.3113 | Yes |
| 349 | NUF2 |  |  | 4782 | -0.847 | -0.3102 | Yes |
| 350 | ASPM |  |  | 4789 | -0.851 | -0.3087 | Yes |
| 351 | TMPO |  |  | 4790 | -0.851 | -0.3060 | Yes |
| 352 | MCM5 |  |  | 4792 | -0.853 | -0.3035 | Yes |
| 353 | CENPN |  |  | 4796 | -0.858 | -0.3013 | Yes |
| 354 | GINS1 |  |  | 4804 | -0.871 | -0.3000 | Yes |
| 355 | LRR1 |  |  | 4805 | -0.873 | -0.2972 | Yes |
| 356 | NCAPG2 |  |  | 4809 | -0.874 | -0.2950 | Yes |
| 357 | CDK4 |  |  | 4810 | -0.874 | -0.2922 | Yes |
| 358 | CDT1 |  |  | 4813 | -0.880 | -0.2897 | Yes |
| 359 | PRIM1 |  |  | 4815 | -0.881 | -0.2871 | Yes |
| 360 | HELLS |  |  | 4819 | -0.882 | -0.2849 | Yes |
| 361 | HSPB11 |  |  | 4820 | -0.882 | -0.2821 | Yes |
| 362 | MCM8 |  |  | 4821 | -0.888 | -0.2792 | Yes |
| 363 | XRCC2 |  |  | 4823 | -0.890 | -0.2765 | Yes |
| 364 | KIF23 |  |  | 4825 | -0.891 | -0.2739 | Yes |
| 365 | MCM3 |  |  | 4834 | -0.901 | -0.2726 | Yes |
| 366 | MKI67 |  |  | 4836 | -0.902 | -0.2699 | Yes |
| 367 | NCAPH |  |  | 4842 | -0.908 | -0.2681 | Yes |
| 368 | KIFC1 |  |  | 4844 | -0.911 | -0.2653 | Yes |
| 369 | PRC1 |  |  | 4846 | -0.912 | -0.2626 | Yes |
| 370 | SMC2 |  |  | 4847 | -0.912 | -0.2597 | Yes |
| 371 | UHRF1 |  |  | 4859 | -0.924 | -0.2590 | Yes |
| 372 | NDUFAF2 |  |  | 4862 | -0.926 | -0.2564 | Yes |
| 373 | DSN1 |  |  | 4864 | -0.929 | -0.2536 | Yes |
| 374 | ORC6 |  |  | 4865 | -0.931 | -0.2506 | Yes |
| 375 | PRR11 |  |  | 4868 | -0.934 | -0.2480 | Yes |
| 376 | JPT2 |  |  | 4871 | -0.938 | -0.2454 | Yes |
| 377 | GMNN |  |  | 4873 | -0.939 | -0.2426 | Yes |
| 378 | CDCA3 |  |  | 4874 | -0.940 | -0.2396 | Yes |
| 379 | CENPM |  |  | 4881 | -0.947 | -0.2378 | Yes |
| 380 | RFC3 |  |  | 4887 | -0.954 | -0.2357 | Yes |
| 381 | GINS2 |  |  | 4897 | -0.962 | -0.2345 | Yes |
| 382 | DUSP23 |  |  | 4899 | -0.964 | -0.2316 | Yes |
| 383 | TACC3 |  |  | 4900 | -0.966 | -0.2285 | Yes |
| 384 | CDCA4 |  |  | 4902 | -0.967 | -0.2256 | Yes |
| 385 | RTKN2 |  |  | 4904 | -0.970 | -0.2227 | Yes |
| 386 | LRRC59 |  |  | 4908 | -0.975 | -0.2202 | Yes |
| 387 | FBXO5 |  |  | 4910 | -0.978 | -0.2172 | Yes |
| 388 | SKA3 |  |  | 4914 | -0.981 | -0.2147 | Yes |
| 389 | FAM111A |  |  | 4915 | -0.981 | -0.2115 | Yes |
| 390 | DNAJC9 |  |  | 4916 | -0.982 | -0.2084 | Yes |
| 391 | PSMC3IP |  |  | 4933 | -0.997 | -0.2085 | Yes |
| 392 | ATAD5 |  |  | 4934 | -0.998 | -0.2053 | Yes |
| 393 | BRCA1 |  |  | 4938 | -1.002 | -0.2027 | Yes |
| 394 | TUBB6 |  |  | 4939 | -1.003 | -0.1994 | Yes |
| 395 | CTNNAL1 |  |  | 4945 | -1.007 | -0.1972 | Yes |
| 396 | MCM4 |  |  | 4947 | -1.011 | -0.1942 | Yes |
| 397 | PSMG1 |  |  | 4955 | -1.025 | -0.1923 | Yes |
| 398 | E2F7 |  |  | 4966 | -1.038 | -0.1911 | Yes |
| 399 | DTL |  |  | 4970 | -1.042 | -0.1883 | Yes |
| 400 | CEP55 |  |  | 4972 | -1.047 | -0.1852 | Yes |
| 401 | CENPW |  |  | 4978 | -1.056 | -0.1828 | Yes |
| 402 | ATAD2 |  |  | 4980 | -1.058 | -0.1796 | Yes |
| 403 | RRM1 |  |  | 4990 | -1.074 | -0.1780 | Yes |
| 404 | HMMR |  |  | 4993 | -1.080 | -0.1749 | Yes |
| 405 | UBE2T |  |  | 5000 | -1.091 | -0.1727 | Yes |
| 406 | MAD2L1 |  |  | 5007 | -1.105 | -0.1704 | Yes |
| 407 | SPC25 |  |  | 5012 | -1.112 | -0.1676 | Yes |
| 408 | FEN1 |  |  | 5014 | -1.113 | -0.1642 | Yes |
| 409 | AURKB |  |  | 5016 | -1.118 | -0.1608 | Yes |
| 410 | TK1 |  |  | 5020 | -1.126 | -0.1578 | Yes |
| 411 | IDH2 |  |  | 5022 | -1.133 | -0.1544 | Yes |
| 412 | CDCA5 |  |  | 5023 | -1.133 | -0.1508 | Yes |
| 413 | TUBA1C |  |  | 5027 | -1.149 | -0.1477 | Yes |
| 414 | KIF2C |  |  | 5028 | -1.150 | -0.1440 | Yes |
| 415 | TOP2A |  |  | 5029 | -1.152 | -0.1403 | Yes |
| 416 | SPHK1 |  |  | 5040 | -1.169 | -0.1386 | Yes |
| 417 | MYBL2 |  |  | 5041 | -1.172 | -0.1348 | Yes |
| 418 | TPX2 |  |  | 5049 | -1.184 | -0.1324 | Yes |
| 419 | CCNA2 |  |  | 5051 | -1.186 | -0.1288 | Yes |
| 420 | NTM |  |  | 5052 | -1.188 | -0.1250 | Yes |
| 421 | DEPDC1 |  |  | 5053 | -1.193 | -0.1212 | Yes |
| 422 | FAM111B |  |  | 5054 | -1.199 | -0.1173 | Yes |
| 423 | NDC80 |  |  | 5055 | -1.200 | -0.1135 | Yes |
| 424 | PCNA |  |  | 5060 | -1.214 | -0.1104 | Yes |
| 425 | MT2A |  |  | 5061 | -1.216 | -0.1065 | Yes |
| 426 | MCM10 |  |  | 5066 | -1.222 | -0.1034 | Yes |
| 427 | ESCO2 |  |  | 5067 | -1.222 | -0.0994 | Yes |
| 428 | BRCA2 |  |  | 5069 | -1.222 | -0.0957 | Yes |
| 429 | CENPK |  |  | 5091 | -1.267 | -0.0960 | Yes |
| 430 | SLC20A1 |  |  | 5094 | -1.277 | -0.0923 | Yes |
| 431 | MTHFD2 |  |  | 5101 | -1.297 | -0.0893 | Yes |
| 432 | UBE2C |  |  | 5106 | -1.314 | -0.0859 | Yes |
| 433 | CDC6 |  |  | 5107 | -1.314 | -0.0817 | Yes |
| 434 | SMS |  |  | 5109 | -1.315 | -0.0777 | Yes |
| 435 | CCDC34 |  |  | 5111 | -1.316 | -0.0737 | Yes |
| 436 | ZWINT |  |  | 5115 | -1.321 | -0.0700 | Yes |
| 437 | CKS1B |  |  | 5121 | -1.338 | -0.0668 | Yes |
| 438 | HJURP |  |  | 5125 | -1.344 | -0.0631 | Yes |
| 439 | C12orf75 |  |  | 5128 | -1.356 | -0.0591 | Yes |
| 440 | RAD51AP1 |  |  | 5133 | -1.375 | -0.0555 | Yes |
| 441 | SLC1A5 |  |  | 5154 | -1.444 | -0.0550 | Yes |
| 442 | PHLDA2 |  |  | 5155 | -1.458 | -0.0503 | Yes |
| 443 | TYMS |  |  | 5156 | -1.477 | -0.0455 | Yes |
| 444 | CENPU |  |  | 5159 | -1.486 | -0.0412 | Yes |
| 445 | DIAPH3 |  |  | 5161 | -1.488 | -0.0366 | Yes |
| 446 | CDK1 |  |  | 5164 | -1.512 | -0.0322 | Yes |
| 447 | PHGDH |  |  | 5170 | -1.528 | -0.0283 | Yes |
| 448 | PLP2 |  |  | 5176 | -1.552 | -0.0243 | Yes |
| 449 | ANLN |  |  | 5183 | -1.577 | -0.0205 | Yes |
| 450 | CLSPN |  |  | 5202 | -1.663 | -0.0189 | Yes |
| 451 | PCLAF |  |  | 5209 | -1.698 | -0.0146 | Yes |
| 452 | HMGA1 |  |  | 5220 | -1.792 | -0.0109 | Yes |
| 453 | HIST1H1B |  |  | 5223 | -1.811 | -0.0055 | Yes |
| 454 | HIST1H1D |  |  | 5231 | -1.871 | -0.0009 | Yes |
| 455 | RRM2 |  |  | 5232 | -1.875 | 0.0051 | Yes |
| 456 | HIST1H4C |  |  | 5249 | -2.081 | 0.0085 | Yes |
Table: GSEA details [plain text format]

  

Fig 2: DOROSZKO\_U3054MG\_CLUSTER\_4: Random ES distribution      
 Gene set null distribution of ES for **DOROSZKO\_U3054MG\_CLUSTER\_4**
